# Deposition of Graphene Oxide on an SPR Fiber Refractometer for Sensor Applications

**DOI:** 10.3390/s23084098

**Published:** 2023-04-19

**Authors:** Maria-Cruz Navarrete, Natalia Díaz-Herrera, Agustín González-Cano

**Affiliations:** 1 Optics Department, Faculty of Physics, University Complutense of Madrid, Ciudad Universitaria s/n, 28040 Madrid, Spain; 2 Optics Department, Faculty of Optics and Optometry, University Complutense of Madrid, Arcos de Jalón 118, 28037 Madrid, Spain

**Keywords:** surface plasmon resonance, fiber optic sensors, tapered optical fibers, graphene oxide

## Abstract

Graphene-based materials have been increasingly incorporated to optical fiber plasmonic sensors due to the peculiar physical and chemical properties of these materials (hardness and flexibility, high electrical and thermal conductivity, and very good adsorption for many substances, etc.). In this paper, we theoretically and experimentally showed how the addition of graphene oxide (GO) to optical fiber refractometers permits the development of surface plasmon resonance (SPR) sensors with very good characteristics. We used doubly deposited uniform-waist tapered optical fibers (DLUWTs) as supporting structures because of their already proven good performance. The presence of GO as an effective third layer is useful to tune the wavelength of the resonances. In addition, the sensitivity was improved. We depict the procedures for the production of the devices and characterize the GO+DLUWTs produced in this way. We also showed how the experimental results are in agreement with the theoretical predictions and used these to estimate the thickness of deposited GO. Finally, we compared the performance of our sensors with other ones that have been recently reported, showing that our results are among the best reported. Using GO as the medium in contact with the analyte, in addition to the good overall performance of devices, permit consideration of this option as an interesting possibility for the future development of SPR-based fiber sensors.

## 1. Introduction

Since its first appearance [1], graphene (G) and its related compounds, such as graphene oxide (GO), have revealed themselves as materials of great interest in many fields [2,3]. This is especially because the electromagnetic and chemical properties of G are very peculiar (hardness and flexibility, high electrical and thermal conductivity, very good adsorption for many substances) [4]. In that sense, it was only a matter of time before the introduction of G and GO into the field of sensing, and more specifically in fiber-optics sensors, would take place [5,6,7]. The most interesting characteristics that the presence of GO in sensing structures can supply include large specific surface area; rich functional groups, which are very convenient for functionalization in chemical sensing [6]; or strong interaction between light and graphene, especially in conditions in total reflection [7]. Using these properties, many sensors have been proposed in fields such as gas sensing [8], humidity measurement for medical and environmental applications [9], or acoustic analysis [10].

Surface plasmon resonance (SPR) sensors are a very good choice in chemical and biochemical sensors as they reach the highest achievable sensitivities while maintaining the advantages of fiber-optics sensors. Many SPR sensors with graphene have also been proposed and several reviews of them can be found in the literature [11,12,13,14]. A whole range of applications of SPR sensors with graphene have been developed in fields such as environmental analysis and protection, biomedicine and healthcare, chemical industry, structural monitoring, food industry, etc. [15,16,17]. This abundance of concepts, schemes, and applications, however, does not imply that the field is exhausted. On the contrary, we are still at the beginning of some very interesting new developments that combine the aforementioned specific characteristics of GO and the good performance of SPR, since in many of the devices reported in the literature, the selected plasmonic structures are not optimal in terms of sensitivity. It is in this area that the work presented here is included. 

As it is well known, the mechanism of operation of SPR sensors is plasmonic resonance, provided that phase matching occurs between the incoming wave and the plasma wave in a structure containing at least one metallic layer, for very specific conditions of wavelength and refractive index of the external medium. In that sense, several structures have been proposed through the years to excite plasmons. One of them that has proven its remarkable performance is the so-called DLUWT (doubly deposited uniform-waist tapered optical fibers) and is one of the best performing plasmonic concepts that has been presented in the literature in recent years [18]. DLUWTs have been extensively tested in the past and are already well known in terms of their properties, and their fabrication process has already been optimized.

Here, we chose to test the new possibilities of GO in SPR sensing, with our objective to maximize these possibilities in terms of sensitivity, versatility, simplicity, and robustness. GO is also interesting for us, compared to G, because it is an oxide and a dielectric, so it, as we will see, can contribute as a third effective layer to the scheme of the DLUWT providing an extra possibility of plasmon resonance tuning. We can even think of it as an option to be used as the sole dielectric layer in combination with metals such as Au or Ag. GO is also very easy to manage and deposit, which is important because the reduction of technological complexity in the fabrication process was one of the goals of the research. As it has also been studied in detail [19], the oxidization of G contributes to the presence of a larger number of oxygen groups, thus providing the possibility of easier functionalization, which is very convenient in SPR structures based on deposited layers to detect specific analytes. It is the first time that a structure such as this, namely GO+DLUWT, has been considered in the literature, so this is a novel and promising approach and opens the possibility of working with other graphene derivatives in the future. 

We show how the addition of GO to these SPR structures influences the tunability of the resonances and increases the sensitivity of the measurements. Since, as it has been said before, GO is interesting in chemical sensing because it provides better interaction with the analytes present in the tested outer medium; it could be a major improvement in the performance of this kind of SPR refractometer. The simplicity of the depicted fabrication process and the fact that we can show good agreement between the simulated and the experimental results also contribute to the idea that counting with graphene materials when considering designs for new plasmonic fiber sensors is a good possibility. 

With respect to the structure of the paper, first we define the concept of the devices and discuss their fabrication process. Then, we show how the simulations predict that the addition of GO to a given DLUWT would translate in a displacement of the resonance, which is one of the key results. Finally, we experimentally characterized the sensors in terms of their response to the variation of the refractive indices, and evaluated their performance, showing a comparison with the equivalent devices without GO deposition, and also use the simulation results to obtain an estimation of the real effective thickness of GO that has been deposited. We conclude with a comparison between the results obtained and those of other similar techniques reported in the literature, showing that ours offer one of the best reported performances.

## 2. Materials and Methods

The structure of the devices is a three-layer deposition on the waist of a tapered fiber, in which GO is deposited on the sensing area of an already existing, previously produced DLUWT. We now briefly depict both the fabrication process of the DLUWTs and the deposition technique used for GO.

### 2.1. Doubly Deposited Uniform-Waist Tapered Optical Fibers

A DLUWT consists of a double deposition on a tapered optical fiber, with a first metal layer, the properly plasmonic material, plus a dielectric one which provides the possibility of tuning the location of the plasmonic resonance at the operating wavelengths and for the desired region of the refractive index of the external medium. The plasmonic resonances show themselves as minima in the spectral transmittance, and these minima shift towards longer wavelengths when the refractive index of the external medium increases due to the presence of an analyte. The sensors, therefore, show a high-level of performance and sensitivity values.

Although the process of fabrication and the evaluation of performance of doubly deposited tapered optical fibers (DLUWTs) has been thoroughly covered on several occasions, [18] for the sake of completeness we will provide some technical information on this topic.

To produce a tapered optical fiber, we used the well-known travelling-burner technique, which is computer controlled and extremely repeatable. The burner oscillates, heating a given region of the fiber which is simultaneously stretched, thus obtaining a given profile for this region in a controlled way, where the section of the fiber was reduced to make the evanescent field of the guided mode accessible [20]. If the taper is fabricated following this process, it can be made quasi-adiabatic since the obtained structure permits smooth transitions between the guided modes. In our case, losses well below 0.1 dB were easily obtained. 

With respect to the dimensions of the taper, along the years we have tested different configurations and construction parameters for DLUWTs. We have found that working with waist lengths of the order of 5 mm and waist thicknesses of 30–40 μm is optimum in terms of robustness and access to the evanescent field (which is the desired goal). 

Once we have the taper, we need to deposit the layers to provide surface plasmon resonance. This is made by physical-vapor deposition, specifically RF sputtering using high purity targets in Ar plasma. The presence of two layers permits us to tune the wavelength of the resonances, adapting the performance of the sensor to the desired range of refractive indices and wavelengths to be measured. The selection of the thicknesses of the layers is made with simulation programs (discussed below), and we were able to achieve plasmon resonances for many different spectral regions and for different ranges of refractive index of the outer medium with this double layer, in particular those of aqueous media with communication fibers which can only be obtained with two layers [21,22].

In general, we exposed the tapers only on one side. As they are curved, this means that the material is not deposited in a homogeneous way. This has been studied by us previously [18]. This inhomogeneity enriches the phenomenology and the possibilities of the devices when compared with the typically used D-fibers. In particular, this is a way of obtaining multiple resonances with a single structure permits us to decrease the dependence of the response of the devices with the polarization in a drastic way, thus simplifying the setup. On the other hand, in the past we have also used a rotating mechanism to achieve full homogeneous 360° deposits, which completely eliminates the dependence on polarization, although providing much wider resonances. In this case, the introduction of the rotating device also complicates the deposition process. Therefore, we have opted for one-sided depositions in general, which are simpler and more convenient.

**Figure 1:**
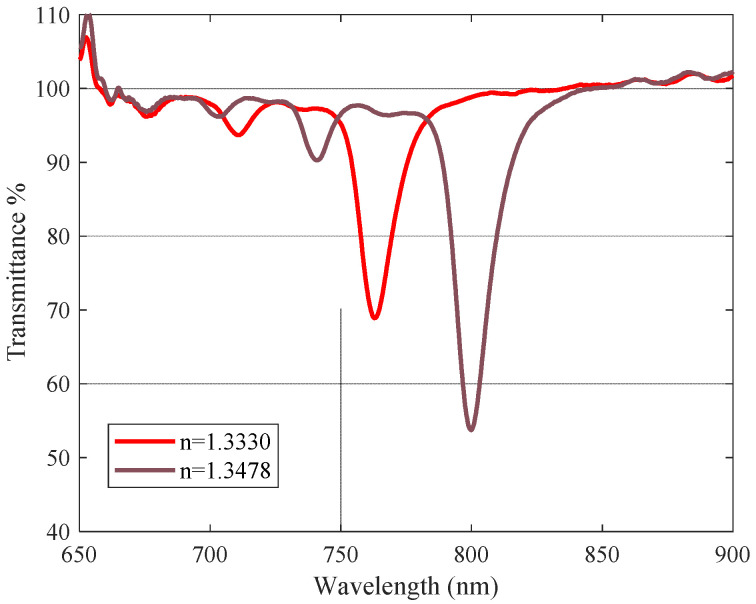
Refractometric characterization curve for a DLUWT with a thickness of 8 nm for the Al layer and 47 nm for the TiO_2_ layer. This corresponds to the employed DLUWT before the GO deposit. We show the variation corresponding to two different refractive indices of the surrounding medium.

In this work, the performance or study of DLUWTs is not the goal, since these are very well-known devices, so we have opted to work with structures that are commonly used by us and with parameters that ensure their correct functioning for the purpose of studying the consequences of the addition of GO. The DLUWTs used in this study were built on a standard silica optical fiber optimized for 830 nm in the travelling-burner setup. We produced tapers with a waist diameter of 40 μm with very low total loss (0.05 dB). Aluminum and titanium dioxide layers were then deposited. The thickness of the deposits was 8 nm for the Al layer and 47 nm for the TiO_2_ layer, in order to provide surface plasmon resonance around 750 nm. A representative plasmon curve obtained with these DLUWTs is shown in Figure 1. 

### 2.2. Graphene Oxide Deposition

**Figure 2:**
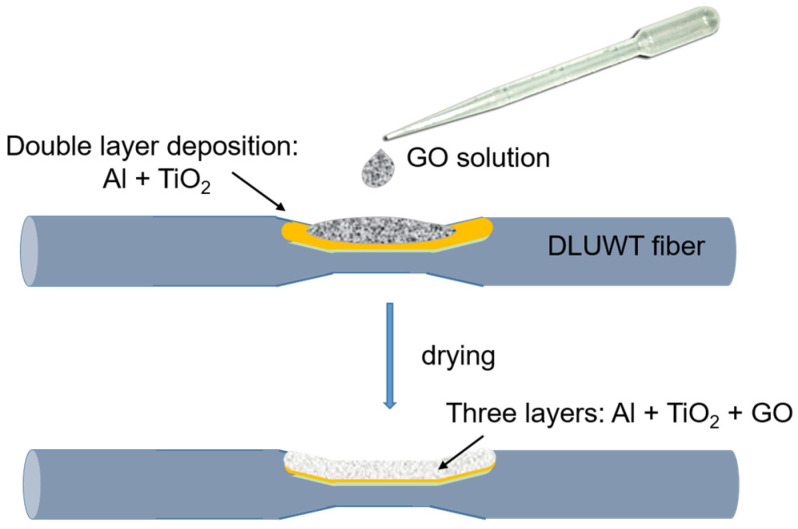
Schematic GO deposition method. A model of the DLUWT is shown. A drop of GO solution is deposited (above) and after drying a third layer is present in the device (below). Further details in the body text.

We opted for a very straightforward method because it was important for us that the fabrication of the devices could be kept as simple as possible. As such, we used the so-called drop-cast method [23]. The results showed that no further complication was required. The technique was used with both naked, undeposited tapered fibers, and with DLUWTs. We added some drops of a solution of water and GO in the narrowed area of the devices, placed on a sample holder and then we let them dry for the desired time interval (Figure 2). The concentration for the GO solution was 0.75 mg/mL. We tested different time ranges and show the results for 10 min.

**Figure 3:**
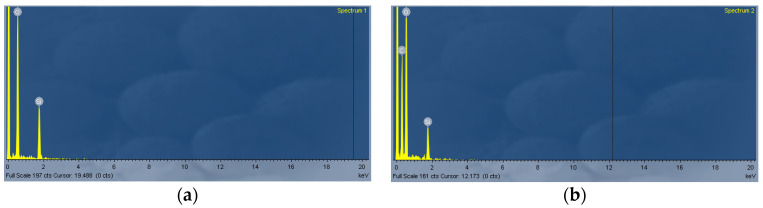
Spectrographic analysis of the surface of (**a**) a naked tapered optical fiber (without any kind of deposit, only silica is present) and (**b**) a GO deposited on a naked taper. A carbon peak associated with graphene appears in this case.

It was important for us to have accurate information on how the deposits adhered to the DLUWT structure. For this, we performed observations with an optical microscope of these deposits. In order to carry out a more precise characterization of the deposits, we analyzed these sensors on a scanning electron microscope (SEM), in the facilities of the CNME (Centro Nacional de Microscopía Electrónica, Madrid, Spain). There, a spectrographic analysis of the composition of the area of interest was performed, comparing it to the composition of an area where no GO had been deposited. In both cases, we could observe peaks associated with silicon and oxygen, which was not surprising since our fiber was made with silica (Figure 3). However, in the deposited area we also observed a different peak associated with carbon, thus confirming that the deposit had been effectively made.

**Figure 4:**
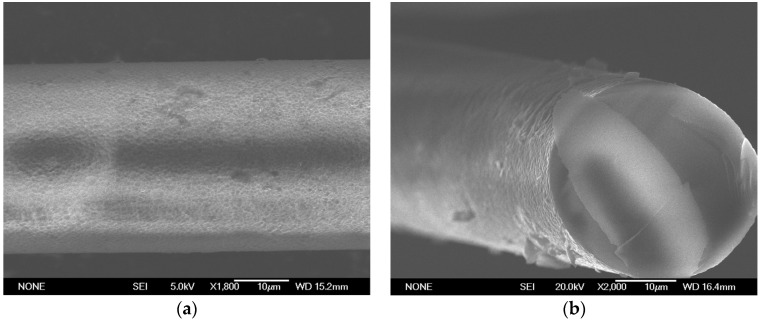
SEM image of the GO deposit on a DLUWT, for an immersion time of 10 min. (**a**) Front view; (**b**) slanted view. The scale and the magnification are shown in the figures (magnifications are ×1800 in (**a**) and ×2000 in (**b**)). The texture associated to the GO deposit can be barely seen because of the presence of the previous deposits of the DLUWT.

Then, the surfaces of the DLUWTs with the GO deposits were also analyzed with SEM. The results can be seen in Figure 4. 

**Figure 5:**
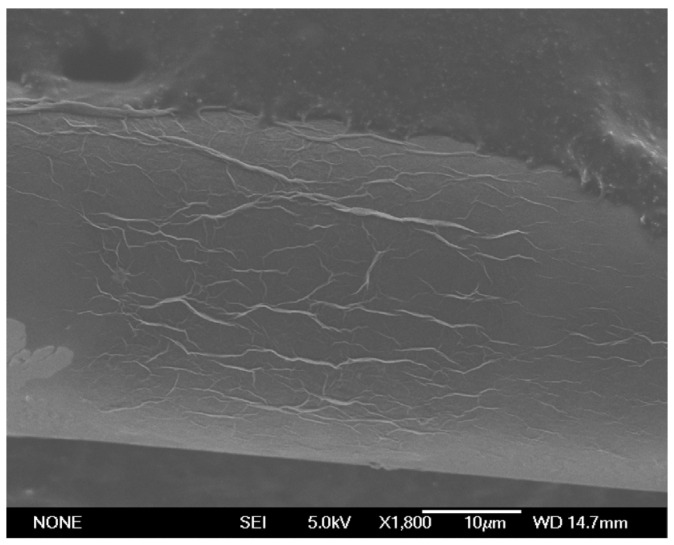
SEM image of the GO deposit on a tapered fiber, for an immersion time of 10 min and similar magnification (×1800) of that of Figure 4. In this case, we did not have any previous deposit on the taper, so the structure associated to GO is more evident.

For a better analysis and understanding of the obtained results, we compared these images with those obtained with a deposit of GO of similar characteristics on a tapered fiber without deposits of Al or TiO_2_ (see Figure 5).

In this case, we can observe, as expected because of the simplicity of the method, that the deposit is not uniform over the entire surface of the tapered optical fiber and typical fold structures of graphene deposits can also be seen. On the other hand, for the DLUWT with GO deposit, the fold structure associated to the GO monomolecular sheets is not so easily perceptible. It is quite logical considering that the relative value of the thickness of GO compared to that of the other layers deposited previously is small. In fact, a more uniform base structure is observed in principle, which is reasonable as the structure underneath the GO layer is clearly evident. This structure is due to the treatment of the surface of the fiber (it was treated with HF for 10 min in order to increase the roughness of the surface, leading to an improvement of the stability of the deposits [24]) as well as the layers deposited previously. Some other accumulations of material associated with the GO deposit can also be observed.

It must be taken into account, however, that the microscope field is small and it is not trivial to get the observed fiber area to the desired zone, which is the one with the greater contact with the GO solution. It corresponds to the waist of the taper but, since we have a narrowing in the fiber, the apparent diameter can lead to distortion. In any case, both transverse and longitudinal non-uniformity of the deposits are expected just from the deposition mechanism, although it is not really relevant for the behavior of our sensors. In this sense, it is not too easy to calculate the typical thicknesses of the deposited GO layers from these measurements, also because the non-uniformity makes it dependent on the area analyzed. However, we can make a rough estimation from the image of the tip of the fiber inclined, knowing the scale of the image. In this way, we can determine that, for the case of deposits of 10 min, as it occurred in the taper of Figure 5, the thickness is of the order of approximately 6–7 nm.

### 2.3. Simulations for the Behaviour of a DLUWT with a Deposit of GO

The simulation of the structures is a very important first step for the fabrication of DLUWTs. Since the thickness of the materials is crucial for the behavior of the devices as plasmonic sensors, and since the presence of the double layer enables us to tune the range of the response in terms of refractometric measurements, we have performed simulations with algorithms that were developed by us to determine how thick must the layers be to provide a good resonance for the refractive indices to be measured and in the spectral range desired, typically in the region of values associated with aqueous media.

The algorithms are based on a simple model that uses a quasi-geometrical approximation, in which we have studied the spectral transmittance of the device structure considering it as a multilayer by obtaining the planar equivalent of the cylindrical waveguide. As such, we can apply the usual matrix treatment for reflectivity calculations in multilayers. It is a straightforward modification of the one presented in [25], that allows us to deal with tapered fibers instead of D-fibers.

In this case, by using these algorithms we can predict the influence that the addition of the GO layer would have in the DLUWTs performance. We then have added GO as an extra medium in these simulations. One very important point is to have accurate inputs for the algorithms. In that sense, we have used the values obtained from the ellipsometric measurements of the optical properties of GO by Schöche et al. for the GO refractive index [26].

**Figure 6:**
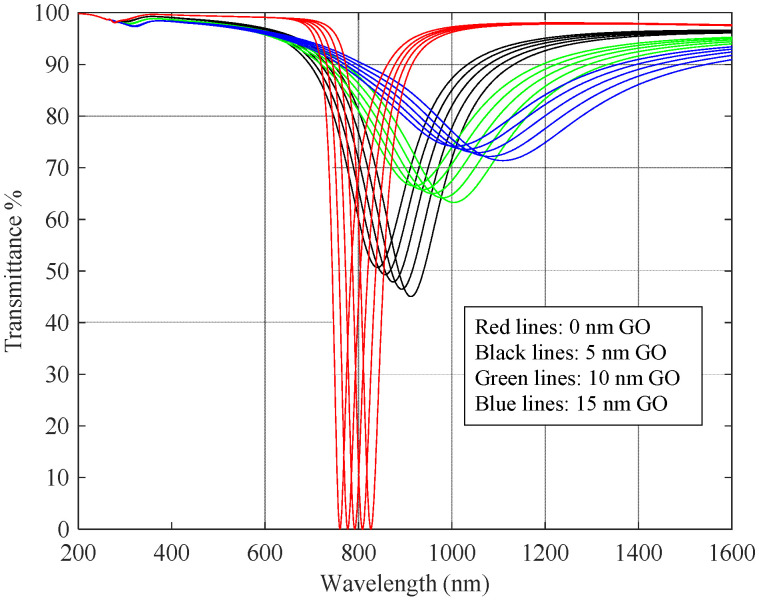
Theoretical spectral transmittance of a GO-deposited DLUWT as the refractive index external medium changes from 1.33 to 1.35, for different graphene oxide layer thicknesses.

Taking into account these values of the refractive index and the thickness of the GO layer, we can calculate the variation in the effective refractive index of the plasmon mode in the structure. This variation will influence the spectral response of the structure to the refractive index of the surrounding medium. This can be assessed by parallel simulations with and without GO. We have studied the expected effect on the spectral transmittance of depositing a thickness between 0 and 15 nm of GO on a DLUWT with the materials and thicknesses already established before. We can see this in Figure 6. 

The minima of these curves are associated with the plasmonic resonance. In the first place, it is observed that GO plays the same role of the dielectric layer traditionally in these sensors, which is to tune the plasmon resonance in the wavelength, displacing the minima to the right when GO is deposited. On the other hand, the increasing thickness of the GO layer makes the plasmon minima become less pronounced and wider. Moreover, the sensitivity seems to be slightly affected by the depositing layer of GO, becoming somewhat higher when the layer becomes thicker. This result is consistent with the fact that, for this kind of sensor, the higher the wavelength working range, the higher the sensitivity obtained. However, if we make a comparison between the theoretical sensitivity of the GO+DLUWT and that of a DLUWT with the plasmon resonance at the same wavelength as the former, we obtain that the sensitivity is slightly higher in the case of adding GO. This in an interesting feature to explore experimentally.

For comparison purposes, we have evaluated the theoretical sensitivities in each case. The sensitivity for the DLUWT sensor is about 3200 nm/RIU. For the devices with GO deposits, the sensitivities increased with respect to that value: up to 3580 nm/RIU for a GO thickness of 5 nm, 4220 nm/RIU for 10 nm, and 5100 nm/RIU for 15 nm. Of course, this kind of simulation does not provide the real operative sensitivities, but, as we will see, the comparison of these values with the experimental ones is interesting.

### 2.4. Experimental Characterization of the GO+DLUWT Plasmonic Refractometer

**Figure 7:**
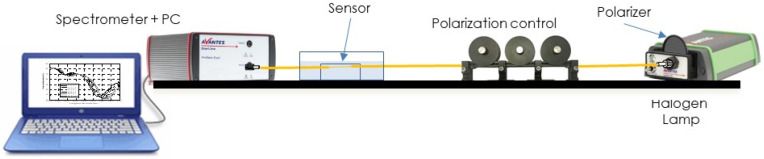
Schematic of the experimental setup for the characterization of the sensors.

For the refractometric characterization of the devices, we have followed our already tested protocol and experimental setup (see Figure 7). 

The light emitted from a halogen lamp (AvaLight-HAL-S-Mini) is directly connected to the polarization-controlling elements (a polarizer and Lefebvre loops) placed before the sensor. At the output, the device is connected to an Avantes spectrometer (AvaSpec 2048-2) to register the transmittance spectra. The sensors are immersed in a mixture of water and ethylene glycol in order to change the refractive index of the external medium, from 1.333 to about 1.36. These refractive index values are measured by means of an Abbe refractometer. We proceeded in a continuous, dynamic way, by adding small quantities of ethylene glycol to the mixture with the sensor submerged. We checked the value of the refractive index and take the measurement, then we increased this value to cover the whole dynamic range. To obtain a descending curve, we subtracted liquid from the mixture, substituting it with water and checking that the values are the corresponding ones. At the end of the process, the sensor was washed with water and no appreciable residue appeared, which was corroborated by the fact that the response of the device was the same that it had been at the beginning of the measuring process.

## 3. Results and Discussion

**Figure 8:**
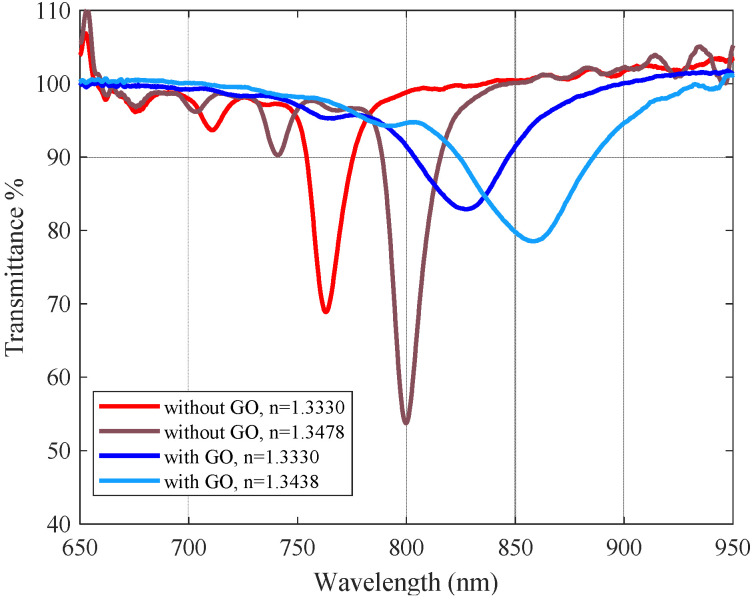
Experimental spectral transmittance against external medium index variations of a DLUWT with (blue lines) and without (red lines) a GO deposition.

In Figure 8, we show representative experimental results of the characterization of the refractometers with and without GO. They corroborate the theoretical simulations, showing experimentally that the GO layer behaves as a dielectric third medium, as the minima shifts towards higher wavelengths, although, as it is always the case with real measurements, in the experimental curves the minima are wider and less pronounced than the theoretical ones.

It should be noted that these “plasmon curves” are calculated by making the quotient between signals with the sensor exposed and not exposed to the surrounding medium with refractive indices capable of exciting plasmons, as is the usual procedure. This can produce apparently misleading results such as “transmittances” over 100%, but we must take into account that these are not transmittances in the exact sense of the term. We have exhaustively studied the problem of the presentation of experimental results in plasmonic fiber sensors in [27]. The presence of GO, as we can see, contributes to some widening of the plasmon dip, mainly due to the inhomogeneity of the deposit, as expected. This is a common feature, but it has no real impact on the measurements because the plasmon is still well defined and we can track it properly.

**Figure 9:**
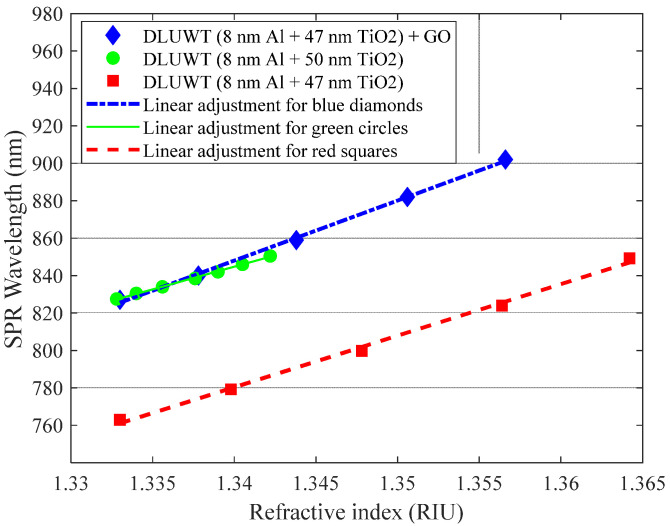
Displacement of the plasmon wavelength due to refractive index variations of the outer medium of a DLUWT with (blue diamonds) and without (red squares) a GO deposition and their corresponding linear adjustments to estimate the sensors sensitivities. It is also shown for comparison (green circles) the curve corresponding to a DLUWT with the resonances in the same wavelength region as those of the GO+DLUWT.

Figure 9 shows the displacement of the minima associated with plasmon resonances as a function of the refractive index. 

As it can be seen, the response is very linear in a wide dynamic range, both with and without GO, which is an interesting feature. With these curves we can estimate the sensitivity for this sensor before and after the deposition of the GO layer. A sensitivity of 3176 nm/RIU was obtained for the device when the GO layer was deposited (blue curve), and 2759 nm/RIU for the DLUWT without the GO layer (red curve). It is, as we see, higher in the case of the sensor with the GO layer. The experimental change of the sensitivity for this device (of the order of 400 nm/RIU) once the GO layer is deposited, is quite similar to that obtained in the simulation for a DLUWT (3200 nm/RIU) and a DLUWT with 5 nm of GO (3580 nm/RIU).

Since the operating wavelength range is higher for the GO+DLUWT with respect to the DLUWT and sensitivities are typically higher for higher wavelengths for these devices, we added another curve (green) to establish whether this improvement in sensitivity is due only to the fact of working at longer wavelengths or whether it is really due to the GO deposit. We compared the sensitivities obtained with a similar sensor whose plasmon resonance appears at those wavelengths. For this purpose, we used a sensor with 8 nm of Al and 50 nm of TiO_2_ and no GO, which shows a plasmon resonance in water at the same position, 827 nm. For this sensor, the sensitivity was 2429 nm/RIU, somewhat lower than in the case of the sensor with a GO deposit (3176 nm/RIU). It must be taken into account that the range for these measurements is smaller and that visually the effect of the sensitivity increasing is not so evident, but if we extrapolate the green curve to higher wavelengths, we see that this effect is much clearer. We can then see that the net increase of sensitivity due to the presence of GO is of the order of 15% in both cases.

Although, as we know, sensitivity is the only of the several parameters to be taken into account when analyzing the performance of a sensor. As such, it is worth noting that the obtained value qualifies as one of the highest among the recently reported ones when considering fiber sensors with GO of different, but related, techniques. We can see this in Table 1, where it is also evident that some of these contributions report quite low sensitivities. In our case, as said before, we take advantage from the fact that DLUWTs are already very sensitive devices. The inclusion of GO, in that sense, is positive and, added to other good characteristics of GO, justify the validity of our proposal. 

**Figure 10:**
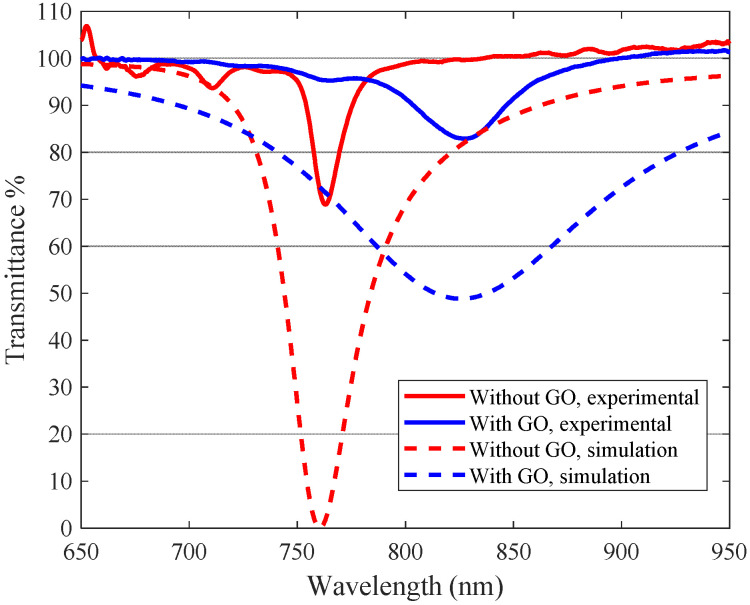
Experimental spectral transmittance in water as an external medium of a DL-UWT with (blue lines) and without (red lines) a GO deposit (solid lines) and the corresponding simulations (dashed lines) to estimate the equivalent layer thickness.

In order to test if the thickness of the GO is of the order of magnitude deduced in Section 2.3, we can also estimate the equivalent thickness of a homogeneous GO layer with the same effect, by considering how much the surface plasmon resonance has been displaced experimentally by the effect of adding the GO layer. To do this, we used the simulation algorithm to calculate the appropriate thickness of GO that would cause this experimentally obtained displacement so the resonances appear as the same wavelengths. This is illustrated in Figure 10. We used the experimental results shown in Figure 8 (note the change of scale) and we added the simulation predictions for the appropriate thickness of GO for comparison. In this particular case, it would be necessary to deposit a GO layer of a thickness of about 4.5 nm, which is somewhat lower than the one estimated from the SEM images but of the same order of magnitude. This thickness value would also be consistent with that estimated from the sensitivity.

As we can see, our experimental results corroborate both the theoretical predictions and the main idea of this paper, namely that GO can (1) be deposited in a simple way in a plasmonic structure of already high performance, (2) that the presence of GO displaces the operative range of the devices toward regions of higher wavelength and higher sensitivity, and (3) that there is an additional, intrinsic increment of sensitivity due to the GO itself. Although some widening of the plasmon dip is unavoidable, this disadvantage is not crucial since the plasmons can still be fully tracked. If we add the fact that the resulting structure, GO+DLUWT, is designed as the basis for the development of varied physical and chemical sensors, the presence of GO as the material directly in contact with the outer medium where the analyte will be present or where the desired parameter to be measured will be associated is very advantageous.

## 4. Conclusions

The objective of the paper was to show how graphene-related materials can be used in the context of plasmonic sensors, and to study how its potential good properties transform the performance of already proven structures with very high levels of performance. As it can be seen from the reported results, we have proven that the addition of GO to plasmonic fiber devices is not only feasible, but simple and straightforward, and that the presence of GO modifies the response of the sensors in an interesting way by increasing their sensitivity and configuring a three-layer system so that can provide extra tunability to the plasmon resonances for the desired sensing range with the devices acting as refractometers. In this way, GO+DLUWT devices repeat the good performance and the desirable characteristics of DLUWTs themselves (roughness, simplicity, high sensitivity, durability, extreme versatility in terms of range of operation, easy integrability, etc.), but with the new feature that the component in contact with the sensed medium (and, therefore, when speaking of chemical sensing, with the analyte) is GO, which, as we know, presents very particular physical and chemical properties such as, in what applies to biochemical sensors in particular, its very good biocompatibility and capacity of adsorption and its high surface–volume ratio. This means that a new field can be explored and that this contribution could be significant in the always moving area of Plasmonics.

The fact that we can use an extremely simple deposition technique for GO and that the experimental results match with the ones predicted by the simulation programs is also to be taken into account, because one of the most important points when designing sensors is the simplicity and cheapness of their fabrication. Another important point is the fact that the dynamics of these kinds of structures, that incorporate first, a tapered fiber, which in itself is a remarkable element in terms of light propagation, and then a triple deposition (metal + dielectric + special material), is interesting from the point of view of basic research.

Although, at this point, the presence of GO in plasmonic sensors has been reported in the literature, it is also true that many of the proposals show rather poor indicatives of performance. Our approach, as has been justified by the comparison with other methods, presents an experimentally measured sensitivity among the highest that have reported in recent times for devices based on similar principles. We are confident that this work can represent a step in the increasing application of graphene to plasmonic devices, which is a subject of the utmost importance, and that our results are relevant and can contribute to the development of these kinds of sensors.

## Figures and Tables

**Table 1:** Comparison of the sensitivity of several configurations incorporating graphene oxide.

Sensing Technique	RI Range	Sensitivity	Ref.
MM fiber + Au + GO	1.335–1.360	2668 nm/RIU	[16]
MM fiber + AuNPs + GO	1.33–1.38	79 nm/RIU	[28]
Hetero-core fiber tip coated Al + HBF + GO	1.3325–1.3745	2280 nm/RIU	[29]
PCS + Ag + GO bilayer film	1.3328–1.3739	3311 nm/RIU	[30]
eFBG + TiO_2_ + rGO	1.40–1.46 (petrol)	6.32 nm/RIU	[31]
SNS + GO (no SPR)	1.3333–1.3846	496.82 nm/RIU	[32]
PCF + Au + GO	1.33–1.37	4649.8 nm/RIU	[33]
DLUWT + GO	1.3333–1.3478	3176 nm/RIU	This work

## Data Availability

Not applicable.
